# Right Sided Intracardiac Thrombosis during Veno-Arterial Extracorporeal Membrane Oxygenation: A Case Report and Literature Review

**DOI:** 10.1155/2019/8594681

**Published:** 2019-01-06

**Authors:** Anusha Ganapati Bhat, Armand Golchin, Deepak Kumar Pasupula, Jaime A. Hernandez-Montfort

**Affiliations:** ^1^Department of Internal Medicine, Baystate Medical Center, University of Massachusetts Medical School, Springfield, MA, USA; ^2^Division of Critical Care Medicine, Baystate Medical Center, University of Massachusetts Medical School, Springfield, MA, USA; ^3^Department of Internal Medicine, University of Pittsburgh Medical Center, Pittsburgh, PA, USA; ^4^Division of Cardiology, The University of Texas Medical Branch, Galveston, TX, USA

## Abstract

Veno-Arterial Extracorporeal Membrane Oxygenation is a common technology of the modern era used as a bridge in severe refractory cardiac and respiratory failure until definitive management is planned. However, early recognition and management of one of the most challenging complications, intracardiac thrombus, continue to remain a conundrum. The incidence of the clinical scenario is very rare. Therefore, due to the lack of literature, there are no guidelines for risk stratification, prevention, or management of intracardiac thrombus. We describe a case of massive pulmonary embolism, who developed a sudden right sided intra-cardiac thrombosis while being optimally anticoagulated on VA ECMO. We also review the literature to describe the pathophysiology, risk stratification, prevention, and management of this rare entity.

## 1. Introduction

Veno-Arterial Extracorporeal Membrane Oxygenation is a technology of the modern era that has progressed quickly and continues to evolve in the world of critical care. Since its initial use in 1970s for postcardiotomy cardiogenic shock, its indications have broadened to involve temporary management of severe refractory cardiac and respiratory failure until a definitive management is planned. But there have been complications associated with them. Based on Extracorporeal Life Support (ECLS) registry report 2017, intracardiac thrombus (ICT) accounts for 5-6% of all the complications of VA ECMO. To our knowledge, most of the ICT is developing while on VA ECMO involved left side or all the chambers of the heart simultaneously. We describe a case of massive pulmonary embolism, who developed a sudden right sided intra-cardiac thrombosis (ICT) while being optimally anticoagulated on VA ECMO. The incidence of the clinical scenario is very rare and reduced even more with the advancement in the ECMO technology. Therefore, due to the lack of sufficient reporting of this clinical entity, there are no guidelines for risk stratification, prevention, or management of ICT. This paper describes the risk factors and pathophysiology for development of intra-cardiac thrombus in a patient treated with VA-ECMO. We will also review the literature describing this rare entity.

## 2. Case Report

A 63-year-old gentleman with a past medical history of alcoholic cirrhosis presented with acute dyspnea, tachycardia, and hypotension to an outside hospital. He was found to have a massive pulmonary embolism bilaterally on Computed Tomography Angiography (CTA) chest. Additionally, there was a sizable thrombus in the right atrium partially extending through patent foramen ovale. Ultrasound Doppler of lower extremities was negative for deep venous thrombosis. The massive PE was managed surgically for immediate stabilization with complete embolectomy followed by patent foramen ovale closure. There was no residual thrombus noted; however, CTA scan was not repeated to evaluate peripheral pulmonary artery thrombosis. Unfortunately, the procedure was complicated by postcardiotomy shock. He failed to be wean from the cardiopulmonary bypass machine and vasopressors (vasopressin, norepinephrine, and epinephrine), thus requiring central Venous-Arterial Extracorporeal Membrane Oxygenation (VA ECMO) support until recovery. Anticoagulation provided perioperatively kept his activated clotting time (ACT) greater than 300s for a few hours. Once ACT was within therapeutic range, he was reinitiated on anticoagulation with a heparin bolus of 50units/kg, followed by continuous infusion at 7.5 units/kg thereafter per ECMO management protocol of the hospital. VA ECMO settings were maintained with a flow of 4.6-4.8 L/min and sweep gas flow rate of 3-4 L/min. His mean arterial pressure was above 65 mmHg and pulmonary arterial pressure was elevated at 29-65 mmHg systolic and 7-25 mmHg diastolic.

On postoperative day 1 (POD 1) he continued to have high flow on VA ECMO, but was noted to have increasing bleed from his nasogastric tube. His hemoglobin dropped from 13.9 gm/dL to 7.5 gm/dL with a hematocrit of 21.6%. His workup was also significant for chronic unchanged thrombocytopenia of 40-50 k/mm3. At this time, systemic anticoagulation was transiently discontinued due to continued bleeding. He was managed with a transfusion of packed red blood cell, platelets, cryoprecipitate, and fresh frozen plasma over a span of 12 hours. In the meantime, he also underwent upper endoscopy, which revealed erosive esophagitis and gastric mucosal erythema, for which he received high dose intravenous proton pump inhibitors. After the completion of the blood products transfusion, his bleeding stopped. Thereafter, he was started on heparin drip at the rate of 10 units/kg/hour and managed with an activated clotting time over 200s, activated partial thromboplastin time 35-75s, and anti-Xa level 0.30-0.70 IU/ml.

**Figure 1:**
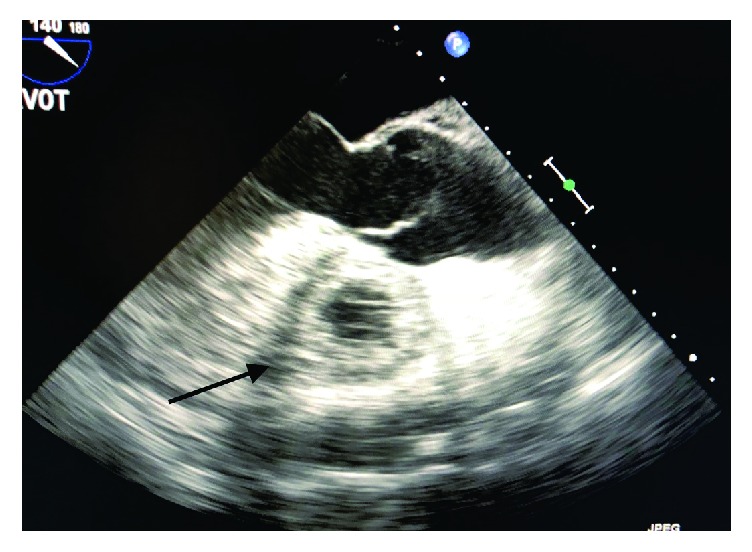
Transesophageal echocardiogram with ME AV LAX view showing extensive thrombus formation in the right ventricle.

**Figure 2:**
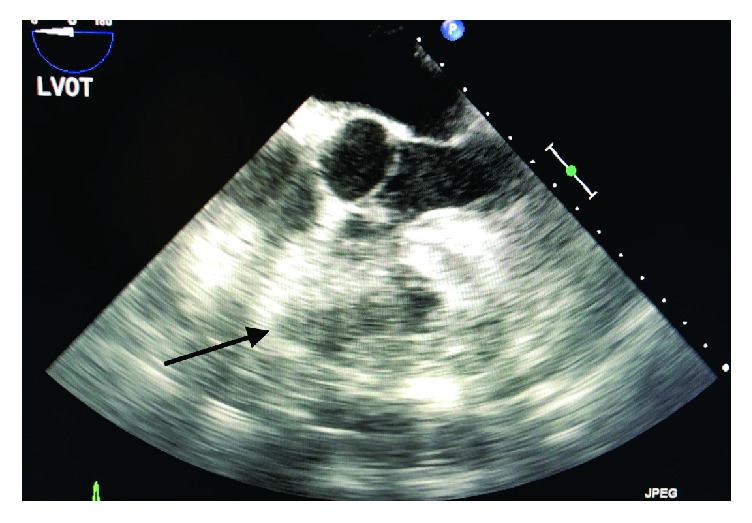
Transesophageal echocardiogram with ME 4CH view showing poor endocardial definition of the right ventricle and the thrombus.

**Figure 3:**
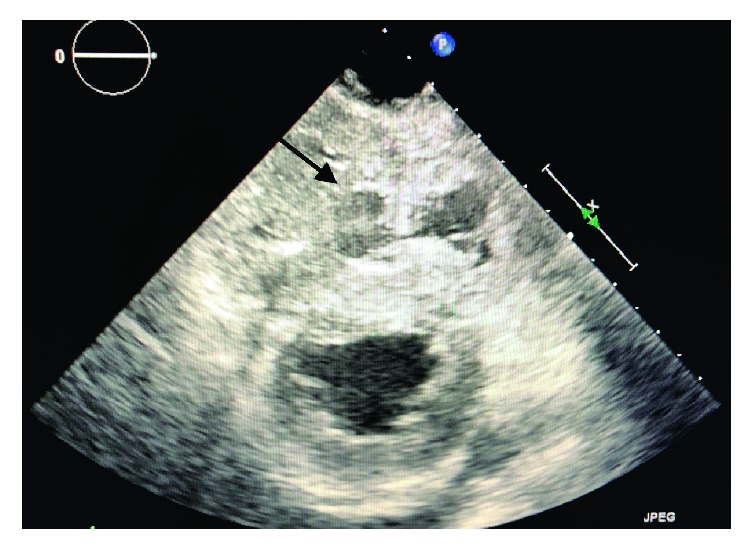
Transesophageal echocardiogram with TG SAX view showing extensive thrombus formation in the right ventricle.

His settings and flow on VA ECMO remained unchanged and he seemed to improve hemodynamically with less requirement of vasopressors in the next 24 hours. He was weaned off epinephrine. His point of care echocardiogram also revealed good cardiac activity without any visible clots. However, towards the end of POD 3, he was noted to develop worsening tachycardia, reduction in ECMO flow to 3.6L/min, and thrombosis of the mediastinal tube. Serotonin release assay for assessment of heparin induced thrombocytopenia (HIT) was negative. A bedside transesophageal echocardiogram revealed global hypokinesis and severe LV dysfunction. Additionally, there was a notably large in situ right ventricular thrombus with a posterior circumferential pericardial clot extending from 3 to 9 o'clock position surrounding the left and right ventricles. He was re-operated immediately with the removal of 1.5L blood and thrombus from the pericardium and mediastinum. There was no obvious site of bleeding noted; however repeat transesophageal echocardiogram suggested reformation of a total right ventricular thrombus, which was confirmed by a transthoracic echocardiogram. The thrombus extended to the main pulmonary artery and its branches. At this point, the family decided to pursue palliative care due to his poor prognosis and the patient expired within the next few minutes (Figures 1, 2, and 3).

## 3. Discussion

ECMO has been used for approximately four decades in intensive care units around the world. Over the years, there have been significant advancements in the technology resulting in improved outcomes and survival rates. Currently, VA ECMO is used for the management of cardiac arrest, cardiomyopathy, myocarditis, and congenital defect and in cardiogenic shock that are difficult to wean from cardiopulmonary bypass (CPB). However, early recognition and management of one of the most challenging complications, intracardiac thrombus, remain a conundrum.

The first case series that introduced ICT as a complication of ECMO was in 1995, where, out of 30 patients on ECMO, 6 developed ICT accounting for 20% incidence rate [1]. Mechanical clots accounted for 15.6% of all the complications and the mortality rate of thromboembolic events while on ECMO has been predicted to be 8.7% [2].

ECMO circuit is a prothrombotic stimulus since it is sensed as a foreign body physiologically. VA ECMO in particular has additional sites of thrombus formation in the heart unlike Veno-Venous ECMO (VV ECMO). Cardiac surgery causes high tissue injury, which may be worsened by further tissue injury during cannulation for vascular access. In addition, blood upon contact with the negatively charged plastic linings connecting ECMO circuit further augments Virchow's triad and sets off the coagulation cascade. In our case, the patient also had a history of alcoholic cirrhosis, which may have hindered coagulation system in the patient with subsequent massive PE in the first place. During the hospital course, his pulmonary arterial pressure (PAP) was high, indicative of evolving pulmonary hypertension as a result of PE, although we cannot ascertain if he already had developed microthrombi in his pulmonary vasculature. Thromboembolism into the blood vessels of the lungs leads to inflammation and interstitial edema subsequently leading to PH, which elevates afterload for the right heart.

Impaired right cardiac output leads to systolic dysfunction, cardiac hypokinesis, and blood stasis, ultimately resulting in ICT, which may be the most likely explanation in our case. In the setting of high prothrombotic risk factors such as coagulopathy from liver disease, additional hypercoagulability induced from multiple blood products transfusion has also been associated with increased odds of thrombosis. A systemic review by Williams et al. established risk factors for the development of ICT following CPB, where patients with surgery for over 2 hours and preexisting congestive heart failure and those undergoing blood products transfusion and antifibrinolytic administration were at a major risk. Of all the blood products, 37.5% of patients developed ICT after platelet transfusion and 27% following plasma transfusion [3].

To prevent ICT, patients are systemically anticoagulated mostly with unfractionated heparin, less frequently low molecular weight heparin, direct thrombin inhibitors, or novel anticoagulation agents, unless heparin is contraindicated. Some instances where thrombus developed while on therapeutic heparin were related to resistance to heparin, which have been partially evidenced to improve with supplemental antithrombin, but definitive evidences are scarce. Although we did not check endogenous antithrombin III level, this intervention is optional in patients undergoing cardiac surgery with CPB, the elderly, and diabetics [4–8]. Also, several advances have been made to the ECMO circuit to minimize hypercoagulability. Use of hollow microfibers to prevent direct blood-gas interactions, preventing artificial surface-blood interactions to reduce hypercoagulability, reducing turbulent flow with low-resistance oxygenators, shorter connections, and minimum circulatory volume are other interventions [9]. These changes in the technology may have reduced incidence of ECMO circuit related thrombosis, but cardiac chambers would still be at risk of forming clots due to low pressure when on VA ECMO. Therefore, systemic anticoagulation is of utmost importance, even though it is challenging as in our case due to increased bleeding potential.

Current protocol for anticoagulation is by combined measurement and assessment of activated clotting time, partial thromboplastin time, and anti-factor Xa levels to adjust anticoagulation therapy to prevent bleeding and clotting imbalance, although in our case they had poor clinical correlatability, which may indicate inappropriate estimation of anticoagulation level.

With anticoagulation, the unfortunate drawback is the risk of bleeding. Disseminated intravascular coagulation, mechanical hemolysis, gastrointestinal hemorrhage, and surgical site bleeds are accountable for up to 50.1% of all the complications from ECMO [10]. But, systemic anticoagulation is one of the key factors to ensure prevention of ICT and requires close monitoring to strike a balance between clotting and bleeding potential. Furthermore, observing pressure gradient changes in the system, timely echocardiograms, and watching for hemodynamic instability will ensure early recognition even when ICT forms.

Once ICT has developed, management options are not evidence based, but based on clinical experience and small studies. Systemic thrombolytic therapy may be fatal due to ongoing bleeds, as in our case, whereas local instillation of thrombolytics may not be helpful due to poor reachability of the medication to the site of ICT due to poor blood flow. Furthermore, inactivation of plasminogen by use of antifibrinolytics during surgery as well as dilutional drop in their levels from volume supplementation for hemodynamic stability may lead to ineffectiveness of tissue plasminogen activator [11].

Current modality used for management of ICT includes surgical thrombectomy, local thrombolysis, or usage of ventricular assist device to ensure adequate forward flow of blood to prevent new thrombus formation from stasis [12, 13]. With ICT on VA ECMO, most patients assume palliative care due to poor outcomes [14]. There is scarce data in the literature to make final conclusion about guidelines for management. But, we can try to improve risk factor predictions and manage patients based on their individual risk stratification. Future research should concentrate on conducting large scale studies for patients on VA ECMO to recognize risk factors and possible successful management of ICT to improve overall mortality outcomes.
